# Sensitivity Analysis of CLIMEX Parameters in Modelling Potential Distribution of *Lantana camara* L.

**DOI:** 10.1371/journal.pone.0040969

**Published:** 2012-07-16

**Authors:** Subhashni Taylor, Lalit Kumar

**Affiliations:** Ecosystem Management, School of Environmental and Rural Science, University of New England, Armidale, New South Wales, Australia; The Australian National University, Australia

## Abstract

A process-based niche model of *L. camara* L. (lantana), a highly invasive shrub species, was developed to estimate its potential distribution using CLIMEX. Model development was carried out using its native and invasive distribution and validation was carried out with the extensive Australian distribution. A good fit was observed, with 86.7% of herbarium specimens collected in Australia occurring within the suitable and highly suitable categories. A sensitivity analysis was conducted to identify the model parameters that had the most influence on lantana distribution. The changes in suitability were assessed by mapping the regions where the distribution changed with each parameter alteration. This allowed an assessment of where, within Australia, the modification of each parameter was having the most impact, particularly in terms of the suitable and highly suitable locations. The sensitivity of various parameters was also evaluated by calculating the changes in area within the suitable and highly suitable categories. The limiting low temperature (DV0), limiting high temperature (DV3) and limiting low soil moisture (SM0) showed highest sensitivity to change. The other model parameters were relatively insensitive to change. Highly sensitive parameters require extensive research and data collection to be fitted accurately in species distribution models. The results from this study can inform more cost effective development of species distribution models for lantana. Such models form an integral part of the management of invasive species and the results can be used to streamline data collection requirements for potential distribution modelling.

## Introduction

Bioclimatic models, species distribution models (SDMs) or ecological niche models (ENMs) are valuable tools that can be used in a variety of applications [1], [2], [3], [4]. One common application of such models is to postulate potential changes in the distribution of invasive species [5], [6], [7]. Such models draw on a species’ distribution data and environmental data to form a species profile that describes how the known presences are distributed in relation to the environmental variables, commonly termed the ‘environmental envelope approach’ [8]. The fundamental principle underlying this approach is that climate is the primary determinant of the potential range of plants and other poikilotherms [9]. The environmental envelope of a species is characterised in terms of upper and lower tolerances and the model is used to produce a habitat map that describes the environmental suitability of each location for the species [8]. This approach has underpinned the development of a range of computer-based systems [2], [10], such as CLIMEX [11] which are designed to model species’ current or their future distributions [1]. In invasive species distribution modelling, the environmental conditions of sites of known occurrence within the species’ native distribution are employed to make projections to other regions to identify potentially suitable areas that can be colonized by non-native populations of the species [5]. Such models provide a useful tool for identifying areas where invasive species could establish and persist and thus are effective for assessing the magnitude of the threat posed.

Although species distribution models are widely used, uncertainties related to the model present many challenges [12] and can have serious implications for the accuracy of the model output. According to [8], one source of uncertainty in species distribution modelling is associated with the assumption that the species is in equilibrium with the environment. Errors resulting from this assumption are most acute when modelling distributions of species recently introduced to new locations. This is particularly pertinent to invasive species which may not be at equilibrium with their current environment in the invaded range because there may be areas where the species is yet to invade, due to limited rates of dispersal, and thus their distributions are still expanding [13], [14]. Residence time has been suggested as an important factor in naturalization and invasiveness [15], [16]. Recent studies have shown that invasive species can inhabit climatically distinct niches after being introduced into a new area [3], [17], [18]. This shift in niche could be the result of biological interactions among species, dispersal rates and evolution of environmental tolerances which are typically not included in the modelling process [3], [19], [20]. Nevertheless, this assumption may be satisfied, to some extent, for invasive species that have been naturalised in their exotic location for long periods of time and are thought to have achieved their full invasive potential [21].

In addition, sources of uncertainty in the model may be addressed by using both native and exotic distribution data for model building. This may produce a model that approximates the potential distribution of the taxa being modelled because the limitations imposed by biotic influences in the species’ native range may be absent in exotic locations, thus allowing it to expand its range beyond its realized Hutchinsonian niche [22]. Furthermore, a larger area of the species range is included in the modelling which introduces a greater number of environmental conditions the species can tolerate [23], thus counteracting the limitations of invasive species niche models based on data from a restricted geographic area as these may have limited applicability for predictive purposes. This has important implications when future projections of species distributions are sought as the true potential range of the invasive species may be underestimated [24].

**Table 1:** The CLIMEX parameter values that were used for *Lantana camara* L. in the baseline model.

Parameter	Mnemonic	Value
Limiting low temperature	DV0	10°C
Lower optimal temperature	DV1	25°C
Upper optimal temperature	DV2	30°C
Limiting high temperature	DV3	33°C
Limiting low soil moisture	SM0	0.1
Lower optimal soil moisture	SM1	0.5
Upper optimal soil moisture	SM2	1.2
Limiting high soil moisture	SM3	1.6
Cold stress temperature threshold	TTCS	5°C
Cold stress temperature rate	THCS	−0.004 week^−1^
Minimum degree-day cold stress threshold	DTCS	15°C days
Degree-day cold stress rate	DHCS	−0.0022 week^−1^
Heat stress temperature threshold	TTHS	33°C
Heat stress temperature rate	THHS	0.001 week^−1^
Dry stress threshold	SMDS	0.1
Dry stress rate	HDS	−0.01 week^−1^
Wet stress threshold	SMWS	1.6
Wet stress rate	HWS	0.01 week^−1^

Parameter uncertainty, related to the data and the methods used to calibrate the model parameters, may also lead to inaccuracies in the model output [12]. A better understanding of the uncertainties associated with model parameterization can be gained by explorations of error using techniques such as sensitivity analyses [25]. Such an analysis is valuable for identifying the parameters that have the most influence on model results [26]. For example, computer based systems that are used for modelling species distributions, such as CLIMEX, may show a higher level of sensitivity to changes in some parameters compared to others. Such differing levels of sensitivity may have substantial impacts on projections of a species’ distribution. A sensitivity analysis of the parameters is necessary to test hypotheses related to the effect of varying climate variables on the distribution of a species as well as providing a better understanding of which aspects of climate have the most impact on populations of a species of interest [27]. Hallgren and Pitman [28] tested the sensitivity of a global biome model to uncertainty in parameter values obtained from the literature. They found that the model was quite insensitive to the majority of parameters but showed considerable sensitivity to some parameters associated with photosynthesis. Crozier and Dwyer [29] modelled range shift of a butterfly following climate change and found that their prediction of range shift was relatively insensitive to changes in individual parameters in their ecophysiological model. Their model was developed using laboratory and field data and was based on the species’ response to temperature.

**Figure 1:**
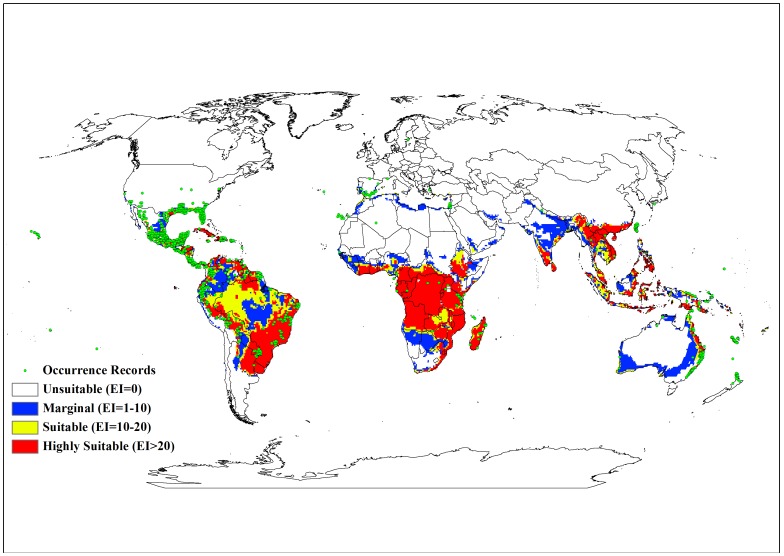
Current and modelled potential distribution of lantana. Data for current global distribution was taken from Global Biodiversity Information Facility 2007.

**Table 2:** Number of herbarium point records within each suitability category.

Suitability Category	No. of Herbarium Points
Unsuitable	14
Marginal	15
Suitable	15
Highly Suitable	174
Total	218

**Table 3:** Impact of sensitivity analysis of the temperature, soil moisture and cold stress parameters on suitable and highly suitable areas.

Parameter Description	Parameter Values	Area of suitable and highly suitable categories (million km^2^)	Difference in Area (million km^2^)	Comparison of EI values with baseline model (R^2^)
DV0 (Limiting low temperature)	4	0.912	+0.341	0.804
	5	0.874	+0.303	0.822
	6	0.836	+0.265	0.844
	7	0.788	+0.217	0.871
	8	0.736	+0.165	0.913
	9	0.655	+0.084	0.968
	**10**	0.571	–	–
	11	0.503	−0.068	0.962
	12	0.443	−0.128	0.890
	13	0.390	−0.181	0.786
	14	0.350	−0.221	0.697
	15	0.304	−0.267	0.588
DV1 (Lower optimal temperature)	18	0.751	+0.180	0.974
	19	0.711	+0.140	0.982
	20	0.687	+0.116	0.988
	21	0.659	+0.088	0.993
	22	0.637	+0.066	0.996
	23	0.613	+0.042	0.998
	24	0.593	+0.022	0.999
	**25**	0.571	–	–
	26	0.544	−0.027	0.999
	27	0.518	−0.053	0.999
	28	0.493	−0.078	0.999
	29	0.466	−0.105	0.998
DV2 (Upper optimal temperature)	26	0.408	−0.163	0.960
	27	0.441	−0.130	0.972
	28	0.472	−0.099	0.985
	29	0.518	−0.053	0.995
	**30**	0.571	–	–
	31	0.631	+0.06	0.994
	32	0.702	+0.131	0.977
DV3 (Limiting high temperature)	31	0.416	−0.155	0.954
	32	0.489	−0.082	0.989
	**33**	0.571	–	–
	34	0.652	+0.081	0.992
	35	0.743	+0.172	0.971
	36	0.765	+0.194	0.968
	37	0.810	+0.239	0.956
	38	0.850	+0.279	0.945
SM0 (Limiting low soil moisture)	0.08	0.623	+0.052	0.997
	0.09	0.600	+0.029	0.998
	**0.1**	0.571	–	–
	0.11	0.546	−0.025	0.999
	0.12	0.520	−0.051	0.997
SM1 (Lower optimal soil moisture)	0.48	0.586	+0.015	0.999
	0.49	0.574	+0.003	0.999
	**0.5**	0.571	–	–
	0.51	0.561	−0.010	0.999
	0.52	0.554	−0.017	0.999
SM2 (Upper optimal soil moisture)	1.18	0.569	−0.002	0.999
	1.19	0.569	−0.002	0.999
	**1.2**	0.571	–	–
	1.21	0.571	0	0.999
	1.22	0.571	0	0.999
SM3 (Limiting high soil moisture)	1.58	0.566	−0.005	0.999
	1.59	0.568	−0.003	0.999
	**1.6**	0.571	–	–
	1.61	0.572	+0.001	0.999
	1.62	0.575	+0.004	0.999
TTCS (Cold stress temperature threshold)	4	0.601	+0.030	0.993
	**5**	0.571	–	–
	6	0.534	−0.037	0.983
THCS (Cold stress temperature rate)	−**0.004**	0.571	–	–
DTCS (Minimum degree-day cold stress threshold)	14	0.581	+0.010	0.998
	**15**	0.571	–	–
	16	0.557	−0.014	0.997
DHCS (Degree-day cold stress rate)	−**0.0022**	0.571	–	–

Values in bold represent the baseline model parameter.

CLIMEX uses the known geographical distribution of a species to infer its climatic response relationships and then projects likely responses to climates in different places and climate change scenarios. The focus, in CLIMEX, is on examining the species’ distribution data to gain a better understanding of the climatic conditions that support the growth or limit the survival of the species [30]. A variety of information types, including direct experimental observations of a species’ growth response to temperature and soil moisture, its phenology and knowledge of its current distribution, are drawn on to model the potential distribution of organisms. In a review of the various climate-based packages designed to estimate potential species distributions, Kriticos and Randall [10] found that ‘CLIMEX was the most suitable climate modelling package for undertaking Weed Risk Assessments because it can support model-fitting to a global plant distribution, includes a climate change scenario mechanism, and provides an insight into the plant’s ecological response to climate’. Furthermore, Webber et al. [31] found that CLIMEX was better placed than two correlative modelling methods (MaxEnt and Boosted Regression Trees) to project a species’ distribution in a novel climate such as a new continent, or under a future climate scenario. Statistical models may describe the geographical distribution of a species precisely, but they are not appropriate for making valid extrapolations to new regions, as is usually necessary with biotic invasions [30]. A further advantage of CLIMEX is that it can reveal when climate alone is not responsible for limiting the geographical distribution of a species and other factors such as biotic interactions may be at work. It is vital to ensure that the parameters are biologically reasonable and the objective is to include all known positive locality records in the parameter-fitting process. Absence in areas that are estimated to be suitable from the climatic conditions associated with presence data may highlight the fact that factors other than climate may be influencing distribution [30].

This study utilised CLIMEX to develop a baseline model for *Lantana camara* (lantana). Lantana is regarded as one of the world’s ten worst weeds [32]. It is invasive in many tropical and subtropical countries outside its native range of Central and northern South America and the Caribbean and its global distribution includes approximately 60 countries or island groups between 35°N and 35°S [33]. It has a range of negative impacts including a reduction in native species diversity, extinctions, decline in soil fertility, allelopathic alteration of soil properties and alteration of ecosystem processes. It has successfully invaded diverse habitats due to its tolerance for a wide range of environmental conditions. In Australia, lantana currently covers more than 4 million ha [33] and costs the Australian grazing industry in excess of $121 million per annum in lost production and management costs [34]. We drew on native lantana distribution data from Central and South America [35] as well as its exotic distribution data from South Africa [36] and Asia [37], [38], [39], [40], [41] for model parameterization to ensure that the complete range of environmental conditions in which lantana may occur was covered. This model was then used to project lantana’s potential distribution, employing the extensive Australian distribution data for model validation. CLIMEX has been utilized by many researchers involved in estimating invasive species’ potential distributions [30], [42], [43], [44], [45], [46] and it allows users to model the potential distribution of organisms based primarily on their current distribution.

**Table 4:** Impact of sensitivity analysis of the temperature, soil moisture and cold stress parameters on validation data.

Parameter Description	Parameter Values	No. of herbarium points in each suitability category			
		Unsuitable	Marginal	Suitable	Highly Suitable
DV0 (Limiting low temperature)	4	9	8	16	185
	5	9	8	16	185
	6	9	9	18	182
	7	9	11	17	181
	8	10	11	19	178
	9	12	13	16	177
	**10**	**14**	**15**	**15**	**174**
	11	22	22	21	153
	12	44	14	18	142
	13	67	14	14	123
	14	84	13	16	105
	15	96	20	14	88
DV1 (Lower optimal temperature)	18	14	5	18	181
	19	14	6	18	180
	20	14	6	18	180
	21	14	8	17	179
	22	14	10	17	177
	23	14	11	18	175
	24	14	15	14	175
	**25**	**14**	**15**	**15**	**174**
	26	14	16	18	170
	27	14	17	24	163
	28	14	17	30	157
	29	15	16	37	150
DV2 (Upper optimal temperature)	26	16	22	32	148
	27	16	19	29	154
	28	16	16	23	163
	29	15	15	20	168
	**30**	**14**	**15**	**15**	**174**
	31	13	14	15	176
	32	12	13	12	181
DV3 (Limiting high temperature)	31	17	20	26	155
	32	16	15	19	168
	**33**	**14**	**15**	**15**	**174**
	34	12	14	16	176
	35	12	13	15	178
	36	12	13	13	180
	37	12	12	13	181
	38	12	11	14	181
SM0 (Limiting low soil moisture)	0.08	13	15	16	174
	0.09	13	15	16	174
	**0.1**	**14**	**15**	**15**	**174**
	0.11	14	16	16	172
	0.12	14	16	17	171
SM1 (Lower optimal soil moisture)	0.48	14	14	16	174
	0.49	14	15	15	174
	**0.5**	**14**	**15**	**15**	**174**
	0.51	14	16	16	172
	0.52	14	16	16	172
SM2 (Upper optimal soil moisture)	1.18	14	15	15	174
	1.19	14	15	15	174
	**1.2**	**14**	**15**	**15**	**174**
	1.21	14	15	15	174
	1.22	14	15	15	174
SM3 (Limiting high soil moisture)	1.58	14	16	15	173
	1.59	14	15	15	174
	**1.6**	**14**	**15**	**15**	**174**
	1.61	14	15	15	174
	1.62	13	14	17	174
TTCS (Cold stress temperature threshold)	4	14	12	15	177
	**5**	**14**	**15**	**15**	**174**
	6	18	16	23	161
DTCS (Minimum degree-day cold stress threshold)	14	12	13	18	175
	**15**	**14**	**15**	**15**	**174**
	16	14	16	20	168

Values in bold represent the baseline model parameter.

The objective of this study was to conduct a sensitivity analysis to quantify the response of lantana to changes in the temperature, soil moisture and cold stress parameters. The main aim was to identify the parameters that were functionally important and thus provide a better understanding of which aspects of climate have a larger impact on lantana distribution. The results should also provide an indication of the parameters that require detailed data collection to be fitted accurately and others that are relatively insensitive to changes and therefore do not require large investments in research and data collection. The implications of this for management are also discussed.

**Figure 2:**
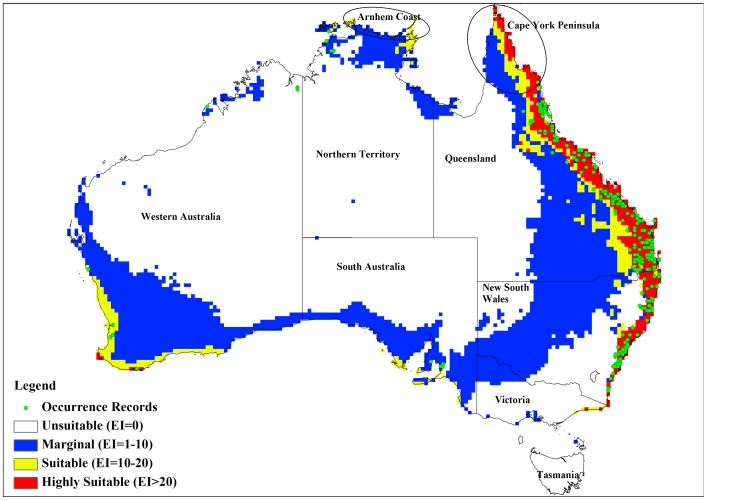
Current and modelled potential distribution for reference climate (averaging period 1961–1990). Data for current Australian distribution is taken from Australia’s Virtual Herbarium.

**Figure 3:**
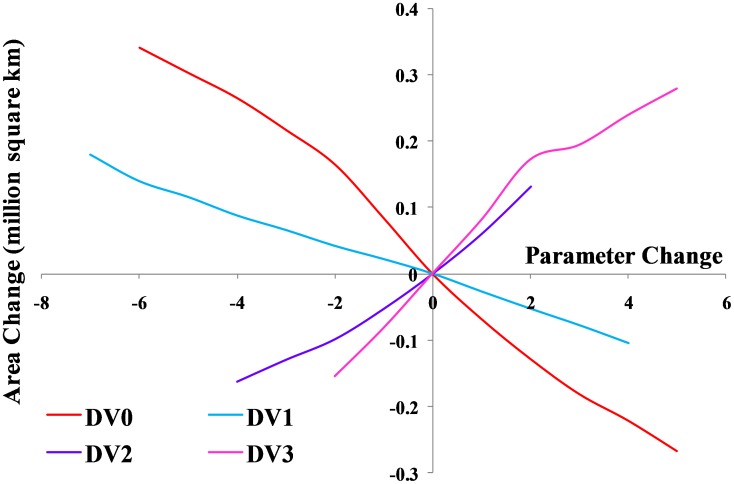
Sensitivity analysis of the temperature parameters in CLIMEX for *L. camara* as change in area of the suitable and highly suitable categories.

**Figure 4:**
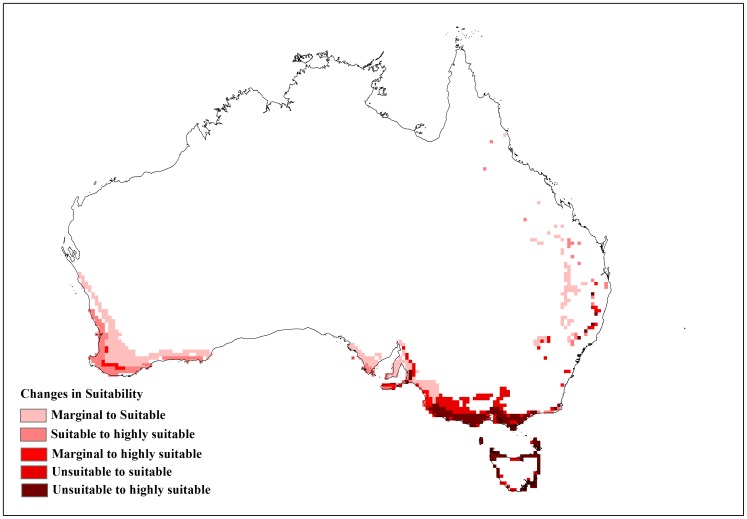
Changes in suitability with limiting low temperature (DV0) at 4°C.

**Figure 5:**
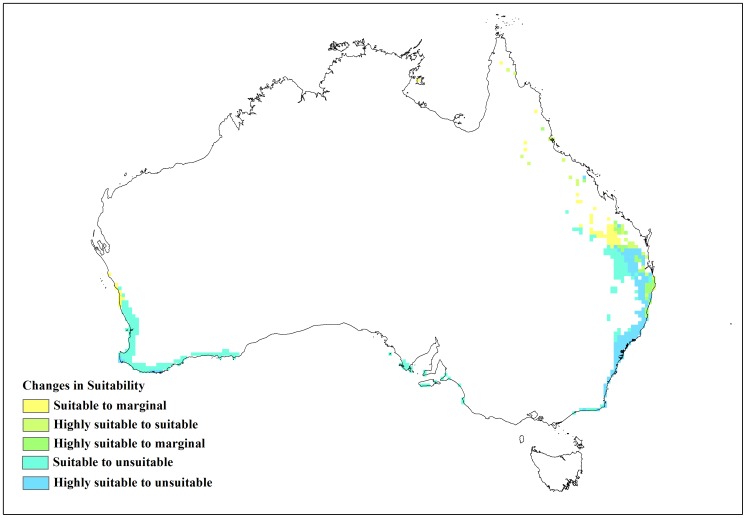
Changes in suitability with limiting low temperature (DV0) at 15°C.

## Materials and Methods

The CLIMEX Version 3 software package [11], [47], [48] works on the basis of an eco-physiological growth model that assumes that a population experiences a favourable season with positive growth and an unfavourable season that causes negative population growth [48]. Parameters that describe a species’ response to climate are inferred from its geographic range [11], [44] and the inferred parameters are then applied to novel climates to project the species potential range in new regions or climate scenarios [49]. The potential for population growth when climatic conditions are favourable is described by an annual growth index (GI_A_) while four stress indices (cold, wet, hot and dry) describe the probability that the population can survive unfavourable conditions [48]. The annual growth index is determined from the temperature index (TI) and moisture index (MI) which depict the species’ temperature and soil moisture requirements for growth. Four parameters, minimum, optimum (lower and upper) and maximum limits to temperature and moisture, respectively, describe the temperature and moisture indices (Table 1). These indices are multiplied to give a weekly growth index which are then averaged to give the annual growth index (GI_A_). Two parameters depict the stress indices, a threshold value and a stress accumulation rate. Stress accumulation during the year is exponential and once the accumulated stress equals 1, the species is not able to persist at the location [48]. Weekly calculations of the growth and stress indices are carried out and combined into an overall annual index of climatic suitability, the ecoclimatic index (EI) which is scaled from 0 to 100. An EI value of zero indicates unsuitable habitat where the species will not be able to survive; marginal habitats are indicated by EI values ranging from 1–10; values ranging from 10–20 can support substantial populations while values above 20 are highly favourable [44].

**Figure 6:**
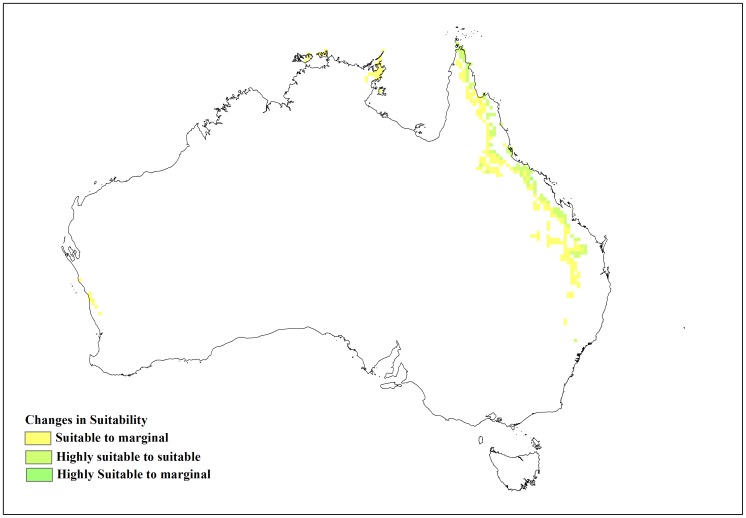
Changes in suitability with limiting high temperature (DV3) at 31°C.

**Figure 7:**
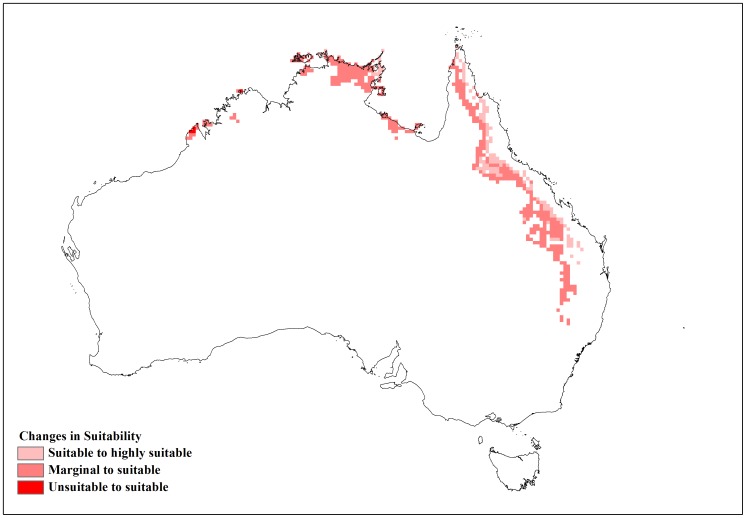
Changes in suitability with limiting high temperature (DV3) at 38°C.

**Figure 8:**
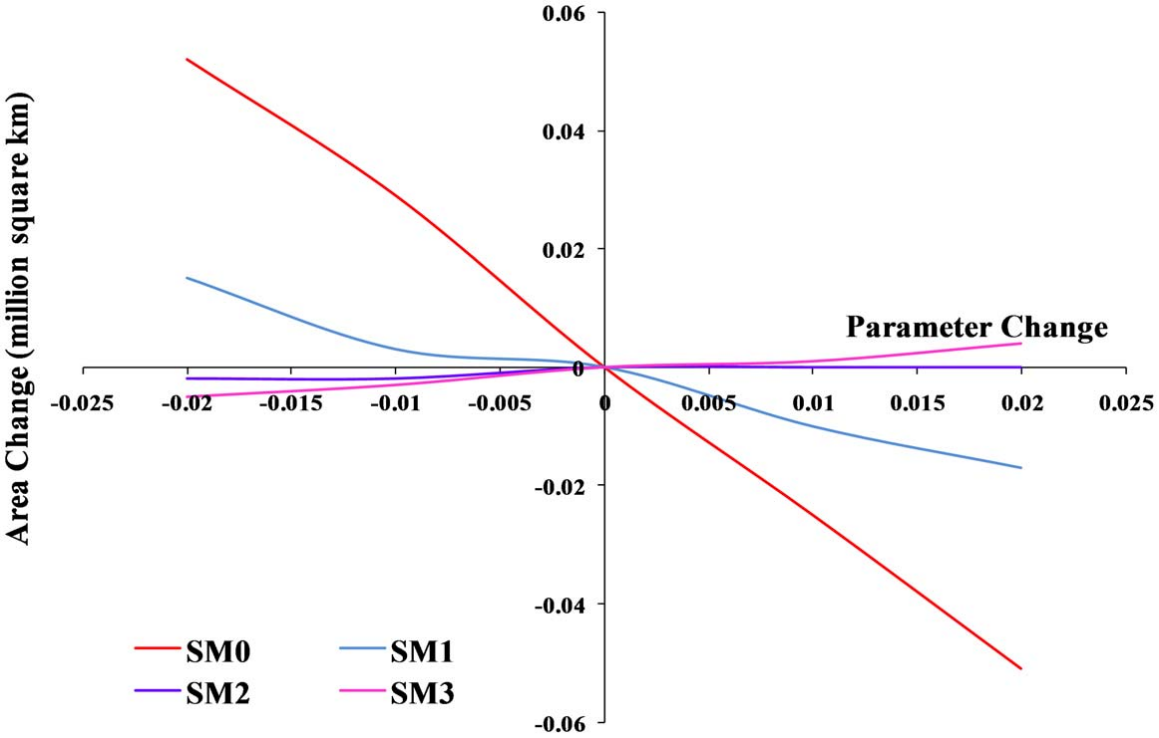
Sensitivity analysis of the soil moisture parameters in CLIMEX for *L. camara* as change in area of the suitable and highly suitable categories.

**Figure 9:**
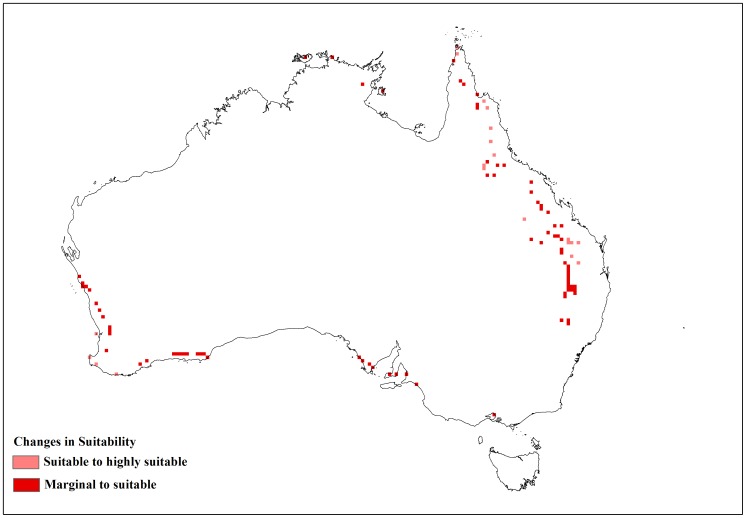
Changes in suitability with limiting low soil moisture (SM0) at 0.08.

**Figure 10:**
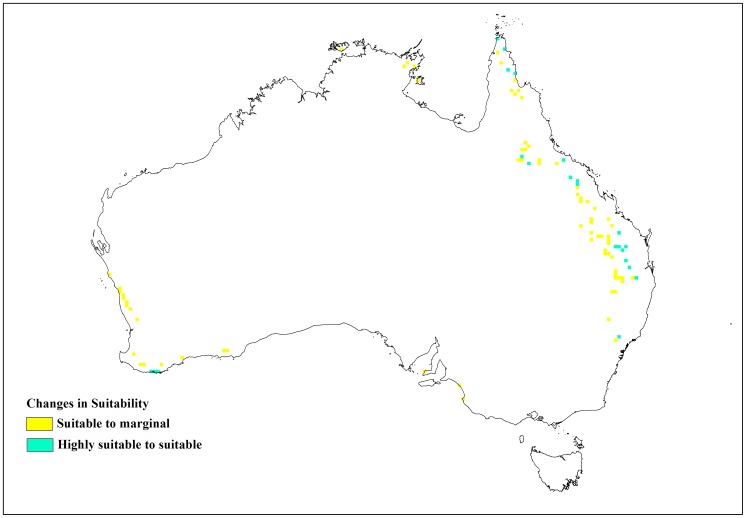
Changes in suitability with limiting low soil moisture (SM0) at 0.12.

Growth and stress parameters were fitted using the methodology described in Sutherst and Maywald [11], Kriticos et al [50] and Chejara et al [51]. A detailed description of the parameters can be found in Sutherst and Maywald [11]. A global meteorological dataset of 0.5° resolution (approximately 50 km×50 km) from the Climate Research Unit (CRU) at Norwich, UK [52] was supplied with CLIMEX. It contained data for a large number of locations across the world and consisted of monthly long-term average maximum and minimum temperatures, rainfall, and relative humidity at 09:00 and 15:00 hours for the period 1961–1990. Initial parameter-fitting was based on this meteorological dataset. Another meteorological dataset for the Australian continent, containing climate data from 1961 to 1990, was utilized for conducting the sensitivity analysis of temperature, soil moisture and cold stress parameters and their impacts on potential lantana distribution in Australia. The Australian dataset included the same five variables as the CRU dataset but at 0.25° (approximately 25 km×25 km) spatial resolution.

The Global Biodiversity Information Facility (GBIF) is a database of natural history collections across the world for a variety of species and it is available for download. Information on lantana distribution was downloaded [35] (Figure 1) for parameter fitting. A total of 4126 records were downloaded but many did not have geolocations and were removed, leaving 2753 records. However, many of these records were repeated several times and were also removed. Thus parameter fitting was based upon 1740 records from the GBIF database. Distribution data from South Africa [36] and Asia [37], [38], [39], [40], [41] were also obtained to assist in the process of parameter fitting. Seasonal phenology data for the southern states of Brazil were employed to fit growth parameters [53], [54]. Although the seasonal phenology observations were restricted to *Lantana tiliaefolia and Lantana glutinosa*, the ecology of these two species are similar to the weedy taxa of lantana, and thus these data were included in model parameterization. An iterative adjustment of each parameter was performed until a satisfactory agreement was reached between the potential and known distribution of lantana in these areas.

A combination of inferential and deductive approaches can be applied in CLIMEX to fit the stress indices [55]. Lantana had a well documented susceptibility to frost [33], [56] and thus this information was drawn upon to inform the choice of cold stress parameters. The cold stress parameters derived from the literature agreed with the distribution information and thus it was concluded that the parameters were satisfactory. An inferential approach was used in the case of parameters that did not have a direct observation of lantana’s response to climatic variables. In this instance, the stress parameters were iteratively adjusted, the model was run and the results compared with our known distribution and phenological data. In the lantana model, the stress parameters were set so that stresses restricted the population to the known southern limits in Buenos Aires and northern limits in India, Nepal and China [37], [38], [39], [40], [41] while allowing it to survive in Kathmandu (27°42′N 85°18′E) [57]. Once the stress parameters were fitted, parameters for the temperature and soil moisture growth indices were adjusted iteratively and model fit was visually assessed until a close match was observed between the projected climate suitability patterns and the observed relative abundance patterns. The objective was to achieve maximum EI values near known vigorous populations and to minimize EI values outside the recorded distribution of lantana. The parameters were checked to ensure that they were biologically reasonable (Table 1). For a detailed explanation of the parameter-fitting procedure, refer to [58]. There is an extensive dataset available from Australia’s Virtual Herbarium (AVH) (http://chah.gov.au/avh/) on lantana distribution in Australia and this was treated as an independent dataset for the purposes of model validation. A total of 635 records were downloaded, many of which were not georeferenced and thus were discarded. A number of the remaining records were duplicates and were also removed, leaving a final set of 218 records. The AVH data were collected between 1902 and 2012. Many of the old records were updated between 1996 and 2009. The final map resulting from the CLIMEX baseline model was validated using this herbarium record data (Table 2). As these locations were not employed in model development, they provided independent validation.

Sensitivity analysis was carried out to quantify the response of lantana to changes in temperature, soil moisture and cold stress parameters. Incremental models were developed from the baseline model to reflect the possible range of these variables that could occur in Australia. During this procedure, the parameter values of the baseline model were kept constant and only one parameter was altered at a time (Table 3). Soil moisture parameters, SM0, SM1, SM2 and SM3 were adjusted with value changes of −0.02, −0.01, +0.01 and +0.02, respectively, from the baseline simulation (SM0 = 0.1; SM1 = 0.5; SM2 = 1.2; SM3 = 1.6). The change value in this case was set quite low because the baseline value for SM0 was already set fairly low. The temperature parameters, DV0, DV1, DV2 and DV3 were also adjusted with value changes of −7, −6, −5, −4, −3, −2, −1, +1, +2, +3, +4 and +5 respectively, from the baseline simulation (DV0 = 10°C; DV1 = 25°C; DV2 = 30°C; DV3 = 33°C). The cold stress temperature threshold (TTCS) was adjusted with changes of −1 and +1 from the baseline value for this parameter (5°C). The cold stress temperature rate (THCS) was kept constant at the baseline value of −0.004 week^−1^ for these two simulations. The minimum degree-day cold stress threshold (DTCS) was varied at −1 and +1 from the baseline model (15°C) while the degree-day cold stress rate (DHCS) was kept constant at the base model value of −0.0022 week^−1^. The stress rates interact with the thresholds by determining how quickly the species accumulates stress when climatic conditions exceed the stress threshold. The stress rates were kept constant because we wanted to assess the effect of changing the stress threshold value on potential distribution.

The adjusted models were re-run after each change in parameter value. In all of the incremental CLIMEX models, stress was only applied outside the range of conditions that were suitable for growth [43], [48]. Both temperature and moisture parameters were always subject to the constraint DV0< DV1< DV2< DV3 and SM0< SM1< SM2< SM3, respectively. The area, in million square kilometres, falling within the suitable and highly suitable categories, was calculated for the baseline and each adjusted model to assess the sensitivity of different parameters (Table 3). Projections from the incremental models were compared with those from the baseline model by plotting EI values for the baseline model against each incremental model from the sensitivity analysis (for each 25×25 km cell) and calculating the R^2^ value. If the parameter that was altered in the incremental model was highly sensitive, we expected large changes in the EI value and therefore a lower R^2^ value. However, if the parameter was not very sensitive, than we expected the EI values of both the baseline and the incremental models to be similar thereby yielding a high R^2^ value (Table 3). The changes in suitability were also assessed by mapping the areas where the suitability had changed in terms of the suitable or highly suitable categories for parameters that showed a high level of sensitivity. Additionally, any changes in the validation data with each parameter change were also assessed by checking any changes in the number of occurrence records that fell within each suitability category (Table 4).

## Results

Figure 1 shows the current recorded global distribution of lantana and the potential global distribution based on EI values from CLIMEX. According to this projected distribution, much of the tropics and subtropics were shown to have suitable climatic conditions for lantana. Large areas of South and Central America, the southern states of USA, Asia, sub-Saharan Africa, Madagascar and the high volcanic Pacific island groups such as Fiji, Vanuatu, Samoa and New Caledonia, among others, had highly suitable climate for the species. These suitable areas were characterised by EI values of 20 and above. Warm temperate areas such as northern New Zealand and southern Mediterranean Europe including Portugal, Italy and Greece were projected as having marginal climatic conditions with EI values between 1 and 10. Although the model of global climate suitability matched the present global distribution of lantana closely, it did not include occurrence records from Mediterranean Europe and Israel. Lantana is mainly grown as an ornamental plant in this region [59] while irrigation plays an important role in the species’ persistence in parts of Israel [60].

Figure 2 shows similar data for Australia and again the current distribution of lantana was largely consistent with the Ecoclimatic Index. In Australia, the model projected much of the eastern coast from Cape York in northern Queensland to southern New South Wales (NSW) as being climatically suitable (Figure 2) with EI values of above 20. However, no occurrence records were found for Cape York Peninsula because, despite a few isolated infestations in this region, lack of human disturbance limits the rate of spread [61]. Small isolated areas in the Northern Territory were also identified as having suitable climate for lantana. Coastal areas along south-west Western Australia were shown to have suitable climate for lantana and this conformed to the actual distribution since small infestations were reported in these areas [33]. Central Australia was projected as being unsuitable (EI value of zero) to marginal (EI values between 1 and 10), mainly due to dry stress. Table 2 shows a high level of correspondence between the potential distribution and the herbarium specimen records for Australia with 189 (87%) records falling within the suitable and highly suitable categories.

The changes to the temperature parameters from the baseline model and their resultant impact on the distribution are illustrated in Figure 3. The baseline model for potential distribution of lantana was very sensitive to changes in DV0, the limiting low temperature, and DV3, the limiting high temperature. When DV0 was set to 4°C (−6 from the baseline model), suitable and highly suitable areas increased by 0.341 million km^2^ and the number of occurrence records that fell within suitable and highly suitable categories changed from 15 to 16 and 174 to 185, respectively, compared to the baseline model. Accordingly, the number of occurrence records in the unsuitable and marginal categories decreased (Table 4). A DV0 value of 4°C caused a southward shift in distribution with larger areas in coastal Victoria and South Australia becoming suitable or highly suitable (Figure 4). A similar trend could be seen with coastal Tasmania. Previously marginal inland locations on the eastern side of mainland Australia also became suitable while others on the eastern coast became highly suitable. More suitable and highly suitable areas could also be seen on the south west coast of Western Australia. However, when DV0 was adjusted to 15°C (+5 from the baseline model), the area in these two categories was reduced by 0.267 million km^2^. The number of occurrence records in the suitable and highly suitable categories decreased from 15 to 14 and 174 to 88, respectively. A substantial change was seen in the unsuitable and marginal category occurrence records with unsuitable records increasing from 14 to 96 when DV0 was set at 15°C. This change in suitability was mainly observed along coastal New South Wales (NSW), coastal Queensland and the south west coast of Western Australia where previously suitable and highly suitable areas became marginal or unsuitable (Figure 5).

Changes in DV3 had an opposite effect compared to DV0 with a −2 (31°C) adjustment of this parameter yielding a reduction in suitable and highly suitable categories of 0.155 million km^2^. Most of these changes could be observed along inland areas of Queensland and coastal Cape York Peninsula (Figure 6). Occurrence records in the highly suitable category decreased from 174 to 155 but suitable category records increased from 15 to 26. Unsuitable and marginal records showed an increase. An adjustment of +5 (38°C) to the baseline DV3 value showed an increase in suitable and highly suitable areas of 0.279 million km^2^. With this parameter change, the number of occurrence records in the highly suitable category increased from 174 to 181 while unsuitable and marginal category records decreased (Table 4). These changes in suitability were seen along inland areas of Queensland extending up to Cape York Peninsula. Increasing DV3 to 38°C also improved suitability in the Northern Territory, particularly along the Arnhem coast (Figure 7). The sensitivity of DV0 and DV3 was also highlighted by the change in EI values between the baseline model and the altered models, generating lower R^2^ values than seen with the other parameter changes (Table 3).

DV1 was modestly sensitive to change. The area in suitable and highly suitable categories changed much more slowly as this parameter was adjusted from the baseline (Figure 3). Lowering DV1 to 18°C increased this area by 0.180 million km^2^. A more substantial change was seen in the occurrence records with highly suitable and suitable category records increasing from 174 to 181 and 15 to 18, respectively. The occurrence records in the marginal category decreased from 15 to 5 while unsuitable occurrence records showed no change. Raising DV1 to 29°C lowered the suitable area by 0.105 million km^2^ and highly suitable occurrence records also decreased from 174 to 150 (Tables 3 and 4). DV2 was more sensitive to change than DV1 as shown by the larger changes in suitable areas and occurrence records when this parameter was altered from the baseline model (Figure 3). A reduction in DV2 to 26°C lowered suitable areas by 0.163 million km^2^. In this case, occurrence records in the highly suitable category decreased from 174 to 148 while suitable category records increased from 15 to 32. Raising DV2 to 32°C also increased suitable areas by 0.131 million km^2^ (Table 3). This change was also reflected in the occurrence records with highly suitable category records going from 174 to 181 while suitable category records decreased from 15 to 12 (Table 4). The higher sensitivity of DV2 was also highlighted by the change in EI values caused by alterations to the baseline model with lower R^2^ values resulting from changes to DV2 compared to alterations to DV1 (Table 3).

Of the four soil moisture parameters, the limiting low soil moisture (SM0) was the most sensitive to changes (Figure 8). An increase of 0.052 million km^2^ in suitable and highly suitable areas was observed when it was adjusted by −0.02 from the baseline simulation. However, this adjustment in SM0 did not lead to large changes in the occurrence records because highly suitable category records remained at 174 with suitable category records only increasing from 15 to 16. Unsuitable records showed a slight decrease. Suitable and highly suitable areas were reduced by a similar amount (0.051 million km^2^) when the baseline SM0 value was adjusted by +0.02. These changes occurred mainly around inland Queensland, NSW, coastal South Australia and the south western corner of Western Australia (Figures 9 and 10). In terms of occurrence records, a +0.02 adjustment to SM0 led to highly suitable category records decreasing from 174 to 171 but suitable category records increasing from 15 to 17. Marginal records showed a slight increase from 15 to 16 (Table 4). A change in the EI values was also observed between the baseline and the altered SM0 models resulting in slightly lower R^2^ values. Alterations to SM1, SM2 and SM3 had little or no effect on lantana distribution (Table 3).

The cold stress temperature threshold was modestly sensitive to changes with an increase of 0.030 million km^2^ in suitable and highly suitable categories when this parameter was adjusted by −1 from the baseline. Conversely, a decrease of 0.037 million km^2^ was observed in these categories when this parameter was varied by +1 from the baseline model (Table 3). The highly suitable occurrence records increased from 174 to 177 while records in the suitable category remained at 15 with a −1 adjustment of the cold stress temperature threshold parameter. No change was seen in the unsuitable category records while the marginal records decreased from 15 to 12. On the other hand, a +1 adjustment of this parameter led to a decrease in highly suitable records from 174 to 161 while suitable category records increased from 15 to 23. Unsuitable and marginal records changed from 14 to 18 and 15 to 16, respectively (Table 4). The minimum degree-day cold stress threshold showed low sensitivity to change with an increase of only 0.01 million km^2^ in the suitable and highly suitable categories when it was changed by −1 and a decrease of 0.014 million km^2^ in the same categories when it was adjusted by +1 from the baseline. The high R^2^ values also reflected the small changes in EI values when these two parameters were changed (Table 3). Occurrence records in highly suitable category showed a small increase (174 to 175) and a larger increase in the suitable category (15 to 18) when minimum degree-day cold stress threshold was adjusted by −1 from the baseline. Unsuitable and marginal category records showed a slight decrease. When this parameter was adjusted by +1 from the baseline, highly suitable category records decreased from 174 to 168 while suitable category records increased from 15 to 20. Unsuitable category records remained the same at 14 and marginal records increased from 15 to 16 (Table 4).

## Discussion and Conclusions

This study shed some light on the precise relationship between climate and the distribution of lantana in Australia. A sensitivity analysis using CLIMEX was informative in identifying specific parameters that had the greatest impact on modelled lantana distribution. The results showed that lantana distribution was highly sensitive to changes in the limiting low (DV0) and limiting high (DV3) temperature as well as the limiting low soil moisture (SM0) parameters. An alteration of DV0 and DV3 had the impact of substantially changing suitable and highly suitable locations for lantana in Australia. A southward shift in distribution was observed when DV0 was lowered from the baseline model. This made new areas such as coastal Tasmania as well as coastal Victoria and South Australia suitable for lantana (Figure 4). According to current distribution data, only isolated infestations have been discovered in Victoria and South Australia [34]. Furthermore, lantana has not naturalised in Tasmania and most of Tasmania is considered unsuitable for its establishment under current climate [62]. Inland areas in the south west corner of Western Australia and some inland locations along the eastern side of the continent changed from unsuitable or marginal to suitable or highly suitable which did not fit with the current distribution data. On the other hand, increasing DV0 from the baseline had the effect of reducing suitable and highly suitable locations, mostly along the NSW and Queensland coast where lantana is prolific (Figure 5). Reducing DV3 from the baseline led to a decrease in suitable areas mainly along the Queensland coast (Figure 6), where lantana is currently abundant. The sensitivity of this parameter may be explained by the recorded susceptibility of lantana to low temperatures and frost [53], [54], [63]. Raising DV3 had the opposite effect of improving suitability for lantana. However, altering this parameter shifted the distribution further inland in Queensland which is not supported by current distribution data. This adjustment in DV3 also made some areas in the Northern Territory suitable, particularly along the Arnhem Coast (Figure 7). This area has had isolated infestations in the past which have been removed and the area is now being monitored to avoid re-infestation [34].

DV1 and DV2 showed some sensitivity to change but they were not as highly sensitive as DV0 and DV3 (Figure 3). The changes in suitability with each parameter change were also reflected in the changes in occurrence records of lantana in Australia (Table 4). In general, where a decrease in suitability was seen in terms of area, the number of records in the highly suitable category decreased while records in the unsuitable and marginal categories increased. In some cases, an increase was observed in the suitable category records which suggest that some highly suitable locations were rendered just suitable by the parameter adjustment. On the other hand, an increase in suitable area was generally matched by an increase in occurrence records in highly suitable and suitable categories with decreases in unsuitable and marginal categories. In this case, a decrease in the suitable category records suggests that some suitable areas changed to highly suitable with the parameter change.

Of the moisture parameters, SM0 was the most sensitive, showing larger shifts in distribution (Figures 8, 9 and 10) when it was altered compared to the other three moisture parameters. SM1 showed a modest level of sensitivity but changes to SM2 and SM3 had very little effect on lantana distribution. Cold stress temperature threshold (TTCS) and minimum degree-day cold stress threshold (DTCS) also showed modest levels of sensitivity to change. The very specific changes to the parameter values during the sensitivity analysis were possible due to an in-depth understanding of this species’ relationship with climate based on the extensive distribution and research data available for lantana both globally and in Australia. Nonetheless, such detailed analyses may not be possible for all invasive species and in particular species which have recently invaded. A different approach may be required for newly arrived invasive species due to the limited understanding of their biology [64].

Predictive modelling of invasive species’ distribution is a useful tool for control and management. These models provide potential distribution maps of invasive species which allows policy makers at both national and international levels to make informed decisions about managing pests. Many predictive modelling techniques, such as CLIMEX, derive information on the climatic requirements of the target species from the geographic distribution data of the species and this informs parameter-fitting during model development. However, the species of interest may be more sensitive to certain climatic factors than others and these varying levels of sensitivity can have serious implications for the predictive modelling of their distribution. Parameters that are highly sensitive to change will have a large impact on the model output compared to relatively insensitive parameters. Sensitivity analysis procedures bring to light relatively more or less important parameters, which can be used to improve data collection plans [65]. Indeed, formal sensitivity analyses have been advocated as the most effective method of evaluating and focussing improvements on model input data [66].

Additional research needs to be conducted to gather more information to fit highly sensitive parameters so that model output may be improved. However, it may not be cost effective to invest in additional data collection for relatively insensitive parameters if the improvement to the model output is modest. Therefore studies that quantify parameter sensitivity and its impacts on model output are useful so that ways of improving confidence in parameter estimates can be identified [64], in order to achieve the most cost effective management strategies for invasive species. In light of this, perhaps the most encouraging finding of this study is that of the ten parameters that were tested, only three appeared to greatly affect the potential distribution of lantana. These were the limiting low temperature (DV0), the limiting high temperature (DV3) as well as the limiting low soil moisture (SM0) parameters. Therefore, in the case of limited resource availability, it would be advisable to invest in fitting these three parameters accurately by researching and incorporating a wide range of alternative data sources [64].

The results of the sensitivity analysis also alert us of the need for caution when using regional and global climate change models on projected lantana distributions, since each of the models and climate change scenarios have a range for both temperature and rainfall variations in the future. This is of particular interest given a projected increase in global average temperatures of 2.4 to 6.4°C based on the Special Report on Emissions Scenarios (SRES) A1F1 fossil intensive scenario [67]. At the lower end of this spectrum is a projected increase of 1.1 to 2.9°C based on the SRES B1 scenario. Such an increase in average global temperature could have serious implications for invasive species such as lantana, particularly given the level of sensitivity this species’ distribution has shown to changes in its upper and lower limits of temperature tolerance. Furthermore, based on the sensitivity that lantana has shown to temperature, and keeping in mind that some of the AVH records that were employed for model validation were collected after 1990, short term changes, particularly between 1990 and 2012, in the continent’s climate may have caused at least a subtle shift in the projected suitability for lantana. This shift may not be reflected in our results because the climate data used in model building and validation were from the period 1961–1990.

Changes in rainfall will also have implications for lantana distribution in Australia, especially if precipitation levels increase, making the arid interior more suitable for lantana than it is currently. Uncertainties surround regional projections of precipitation in light of climate change [67]. However, the general regional expectation is a likely decrease in annual precipitation in most parts of Australia, with a 5–10% decrease in annual rainfall under the B1 scenario and a 20–40% decrease in annual rainfall under the A1F1 scenario [68]. Likely increase in extremes of daily precipitation throughout Australia and a likely increase of drought are also projected along with extreme weather events such as heat waves [67]. Such changes in precipitation levels will have implications for lantana distribution, especially given its sensitivity to changes in the lower limits of soil moisture (SM0). The sensitivity that lantana distribution has shown to different parameters indicates that the results from a modelling exercise will vary depending on which SRES scenario is used. The results from this study should alert modellers to the risk of using just one scenario for modelling lantana distribution. Therefore, management decisions should be based on distribution results from an ensemble of scenarios.

The main assumption within CLIMEX is that climate is the primary determinant of the geographical distribution of a species. Therefore, non-climatic factors such as dispersal potential, biotic interactions, soil type, land-use and disturbance activities are not included explicitly in the modelling process. However, these factors can be incorporated after the climate modelling has been carried out [51]. Furthermore, the inclusion of both native and exotic distribution data should capture any effects from the release from natural enemies [69] that are apparent in lantana’s exotic range. This gives a clearer picture of its fundamental niche [70].

Lantana has a very widespread distribution in Australia and total eradication is not feasible given the current management resources and technologies [34]. Therefore, methodologies that refine the data requirements of potential distribution modelling tools for lantana are worthwhile. This study used sensitivity analysis to identify CLIMEX parameters and consequently aspects of climate that had the most influence on the potential distribution of lantana in Australia. This approach can be used to streamline data collection requirements for potential distribution modelling.
